# Inferring novel gene-disease associations using Medical Subject Heading Over-representation Profiles

**DOI:** 10.1186/gm376

**Published:** 2012-09-28

**Authors:** Warren A Cheung, BF Francis Ouellette, Wyeth W Wasserman

**Affiliations:** 1 Bioinformatics Graduate Program, Centre for Molecular Medicine and Therapeutics at the Child and Family Research Institute, University of British Columbia, 980 W. 28th Ave, Vancouver, V5Z 4H4, Canada; 2 Department of Cells and Systems Biology, Ontario Institute for Cancer Research, University of Toronto, 101 College Street, Toronto, M5G 0A3, Canada; 3 Department of Medical Genetics, Centre for Molecular Medicine and Therapeutics at the Child and Family Research Institute, University of British Columbia, 980 W. 28th Ave, Vancouver, V5Z 4H4, Canada

## Abstract

**Background:**

MEDLINE^®^/PubMed^® ^currently indexes over 18 million biomedical articles, providing unprecedented opportunities and challenges for text analysis. Using Medical Subject Heading Over-representation Profiles (MeSHOPs), an entity of interest can be robustly summarized, quantitatively identifying associated biomedical terms and predicting novel indirect associations.

**Methods:**

A procedure is introduced for quantitative comparison of MeSHOPs derived from a group of MEDLINE^® ^articles for a biomedical topic (for example, articles for a specific gene or disease). Similarity scores are computed to compare MeSHOPs of genes and diseases.

**Results:**

Similarity scores successfully infer novel associations between diseases and genes. The number of papers addressing a gene or disease has a strong influence on predicted associations, revealing an important bias for gene-disease relationship prediction. Predictions derived from comparisons of MeSHOPs achieves a mean 8% AUC improvement in the identification of gene-disease relationships compared to gene-independent baseline properties.

**Conclusions:**

MeSHOP comparisons are demonstrated to provide predictive capacity for novel relationships between genes and human diseases. We demonstrate the impact of literature bias on the performance of gene-disease prediction methods. MeSHOPs provide a rich source of annotation to facilitate relationship discovery in biomedical informatics.

## Background

A key focus of genomic medicine is the identification of relationships between phenotype and genotype. Genome-wide association studies and exome/genome sequencing can reveal hundreds of candidate genes that may contribute to human disease. Given such a set of candidate genes, the prioritization of these genes for functional validation emerges as a key challenge in biomedical informatics [1]. Much focus has been placed upon the development of methods for the quantitative association of genes with disease [2].

Across biomedical research fields, scientific publications are the currency of knowledge. One near-universal tool of life scientists to access this 'bibliome' is the MEDLINE^®^/PubMed^® ^bibliographic database of the US National Library of Medicine (NLM), an actively maintained central repository for biomedical literature references [3]. As of 2010, over 18.5 million citations have been indexed by MEDLINE^®^, at a modern rate exceeding 600,000 articles per year. Researchers face increasing difficulty navigating the growing body of published information in search of novel hypotheses. Encapsulating the bibliome for a disease or gene of interest in a form both understandable and informative is an increasingly important challenge in biomedical informatics [4,5].

MEDLINE^® ^provides data structures and curated annotations to assist scientists with the challenge of extracting pertinent articles from the bibliome of a biomedical entity. In an ongoing process, curators at the NLM identify key topics addressed in each publication and attach corresponding Medical Subject Headings (MeSH) [6] terms as annotations to each publication's record in MEDLINE^®^, covering over 97% of all PubMed-indexed citations. The National Center for Biotechnology Information (NCBI) PubMed portal utilizes the annotated MeSH terms to empower search of the citation database, extending the reach of users beyond naïve word matching to topic matching. As one of the constellation of NCBI resources, MEDLINE^®^/PubMed^® ^citations are further linked to gene entries in Entrez Gene where appropriate, with over 450,000 MEDLINE^®^/PubMed^® ^citations linked to an Entrez Gene entry for a human gene.

The analysis of gene annotation properties and gene-related literature is a core challenge within computational biology. Biomedical keywords for properties of genes, drawn from structured vocabularies, have been identified from unstructured gene annotations [7,8], as well as directly from the primary literature [9-11]. Sets of genes can be analyzed to extract common annotated biomedical properties[12]. Assigned descriptive terms can be visualized as 'tag clouds' [13,14]. Comparison of gene annotation profiles can group genes - expanding protein-protein interaction and phenotype networks, deriving regulatory networks and predicting other gene-gene relationships [15-20]. Annotation analysis enables prioritization of candidate genes in genetics studies [10,21-23], and, when integrated with other information sources, predicts novel properties of genes [24,25]. Existing tools and techniques demonstrate the value, and suggest a high potential impact, of annotation analysis. Significant research opportunities remain to improve annotation and annotation-based analysis methods.

The development of computational disease information resources has run parallel to the aforementioned gene-based efforts. Controlled vocabularies for medical descriptions [26,27] and disease-specific annotations [28,29] are emerging to facilitate medical information systems. Analysis of biomedical annotations associated with disease literature, as well as networks of gene-disease association, have been constructed to investigate the common biological aspects underlying diseases [9,30]. In tandem with the curation of MEDLINE^® ^by the NLM, a disease category of the Medical Subject Headings has been developed over 50 years, providing an extensive inventory of medical disorders. By 2011, 4,494 MeSH disease terms had been established.

Key to accelerating the identification of gene-disease relationships is the development of systematic approaches to quantitatively represent bibliometric information and infer functionally important relationships between entities. We have previously introduced MeSH Over-representation Profiles (MeSHOPs) as a convenient tool for constructing quantitative annotations for sets of papers in MEDLINE^® ^where each paper refers to the same entity (such as a gene or a disease) [31]. To demonstrate the fidelity of the MeSHOP knowledge representation at measuring features important for prediction, we generate the MeSHOPs for human genes and diseases, and compare these MeSHOPs to predict novel associations. Predictive performance for gene-disease relationships is validated against co-occurrence in future publications and curated databases. Comparing MeSHOPs is demonstrated to be an effective way to identify novel relationships between genes and diseases.

## Results

### Generation of MeSHOPs

**Figure 1:**
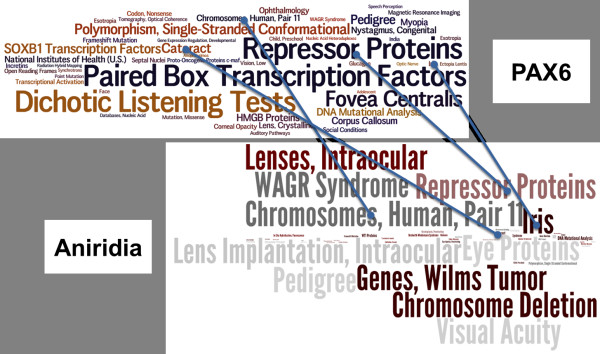
**Comparing gene and disease MeSHOPs**. A graphical representation of the comparison of the MeSHOPs for the human gene *PAX6 *and the disease aniridia. The most strongly associated terms for each profile are presented as a word cloud, scaling the size of each term with the degree of association. Blue lines link shared terms between the profiles - the similarity scores quantitatively evaluate the difference between the profiles by comparing all shared terms between profiles.

Disease and gene MeSHOPs provide a concise quantitative representation of the biomedical knowledge associated with an entity (Figure 1). For this study, two large classes of entities were analyzed - the human genes in Entrez Gene and the diseases specified formally within MeSH. MeSHOPs were generated for the classes 'disease' and 'human gene' by assessing the set of all linked MEDLINE^®^/PubMed^® ^records for each entity.

All human genes present in Entrez Gene were considered (38,604 in Entrez Gene 2007). Two sources for gene-article linkages from Entrez Gene were evaluated: Gene Reference Into Function (GeneRIF) and *gene2pubmed*. GeneRIF is a curated set of links from Entrez Gene to MEDLINE^®^/PubMed^® ^citations provided by annotators at the NLM and supplemented by validated public submissions that specifically refers to a described function of the gene [32]. These allow us to generate profiles based on articles highly relevant to the gene of interest, looking specifically at the subset of articles addressing the function of the gene. *gene2pubmed *is a set of links to MEDLINE^®^/PubMed^® ^articles relating to the gene, generally broader in scope than GeneRIFs, combining information from a panel of public databases. Due to its more general nature, *gene2pubmed *provides gene links for a larger proportion of the human genes, and links more articles for each gene as it is not limited to articles specifically addressing the function of the gene. We use these two sets of links to examine the effect of the quantity and specificity of gene-associated literature on prediction performance. GeneRIFs link 11,750 human genes to 142,396 articles. *gene2pubmed *links 26,510 human genes to 226,615 articles. The two MeSHOP gene article linkage collections are both used in the subsequently described validation.

Disease MeSHOPs were generated directly from MEDLINE^® ^via the curator-assigned MeSH disease terms. To generate MeSHOPs for diseases, all terms from the disease category - MeSH category C [33] - were used; a set composed of 4,229 unique terms in MeSH 2007 linking to over 8 million articles (of 16 million MEDLINE^®^/PubMed^® ^articles).

### Quantitative comparison of gene and disease MeSHOPs for prediction of future co-occurrence in research publications

We hypothesize that a disease is likely to be associated with a gene if the disease MeSHOP is highly similar, under a quantitative profile comparison metric, to the gene MeSHOP. For example, a disease with a functional relationship to a gene may share MeSH terms between profiles, such as localization, metabolic pathways, cellular processes and symptoms, even if no links between the gene and the disease have been previously reported in the literature. When many biomedical terms are common between two profiles, the likelihood for a future association between the entities profiled is expected to increase.

Gene-disease relationship predictions using MeSHOPs from 2007 are validated here against gene-disease co-occurrences that appear in subsequent MEDLINE^® ^(that is, using data not represented in the MeSHOPs). A validated prediction means one or more articles referring to both the gene and the disease was published during a subsequent time period as reported in a future MEDLINE^® ^release (2009 or 2010). Two overlapping validation sets (2007 to 2009 and 2007 to 2010) were extracted: (i) 95,845 novel gene-disease co-occurrences for gene-article mappings from *gene2pubmed *for 2007 to 2009; (ii) 183,407 novel gene-disease co-occurrences for mappings from *gene2pubmed *for 2007 to 2010; (iii) 95,085 novel gene-disease co-occurrences for gene-article mappings from GeneRIF for 2007 to 2009; and (iv) 169,723 novel gene-disease co-occurrences for mappings from GeneRIF for 2007 to 2010. This approach is similar to the validation scheme presented in [34].

Using these validation sets, we evaluate scoring methods by computing the receiver operating characteristic (ROC) curve for predictions from analysis of the baseline 2007 data and reporting the area under the ROC curve (AUC). MeSHOP comparisons are defined as predictions of future disease-gene co-occurrence if a similarity score exceeds an applied threshold. To calculate the ROC curve, we classify the novel gene-disease co-occurrences appearing in the future gene MeSHOPs as 'true positives', and all other gene-disease pairings as 'true negatives', and for each possible threshold, we measure the sensitivity and the false positive rate. An ideal prediction method will produce an AUC score of 1, while random predictions are expected to generate an AUC score of 0.5.

### Gene and disease predictive bibliometric baselines

**Figure 2:**
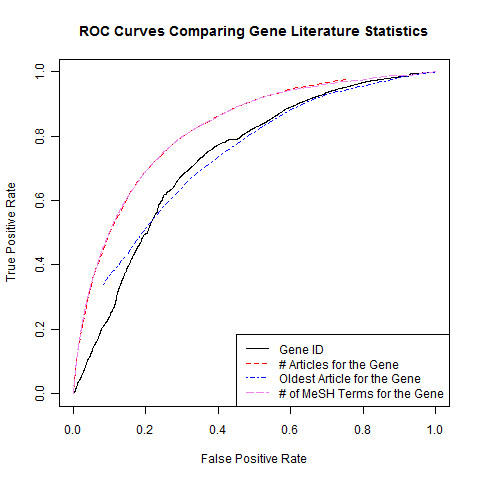
**Comparison of performance of gene characteristics**. ROC curves are shown comparing predictive gene characteristics. Characteristics are computed from a 2007 Entrez Gene dataset and the MEDLINE^® ^Baseline 2007, predicting against all new disease terms associated to gene MeSHOPs between February 2007 and April 2010.

There is little quantitative information about baseline performance against which to compare gene-disease association prediction methods. Intrinsic characteristics of genes (for example, Entrez Gene identification number) were assessed for capacity to predict future gene-disease term co-occurrence. For these controls, scores were obtained for quantitative characteristics of each gene. The scores represent gene-specific properties and do not account for disease properties; gene rankings are therefore the same for each disease in this baseline assessment. Likewise for the one examined intrinsic disease characteristic (number of MeSH terms), the diseases are ranked without regard to any specific gene. Using these baseline rankings, the AUC score was calculated for each indicated characteristic (Figure 2). Gene-specific characteristics evaluated were: percentage of G/C mononucleotide content of the primary RefSeq transcript, total number of associated cDNA sequences reported in Entrez Gene, RefSeq transcript length, genomic length (from the annotated Ensembl gene/transcript start to end) and the Entrez Gene identification (ID) numbers. GC content, number of transcripts, transcript length and genomic length produced random AUC scores (approximately 0.5; Table 1).

**Table 1:** Performance of gene characteristics at predicting association with disease

	*gene2pubmed*			GeneRIF		
	
Scoring method	Validation (02/2007-01/2009)	Validation (02/2007-04/2010)	CTD validation (11/2008)	Validation (02/2007-01/2009)	Validation (02/2007-04/2010)	CTD validation (11/2008)
Percentage GC content	0.50	0.50	0.51	0.50	0.50	0.51
Number of transcripts	0.53	0.53	0.55	0.51	0.51	0.53
Transcript length	0.51	0.52	0.50	0.52	0.52	0.53
Genomic length	0.52	0.52	0.50	0.51	0.51	0.52
Gene ID	0.73	0.71	0.78	0.64	0.63	0.69

Characteristics were compared against the 02/2007-11/2008 validation sets using *gene2pubmed *and GeneRIF gene references, as well as the 11/2008 Comparative Toxicogenomics Database (CTD) validation set. Gene characteristics were extracted from EnsEMBL. We compare the performance of these characteristics at predicting new gene-disease relationships in our validation sets (for the genes with mapped characteristics).

Strikingly, Entrez Gene ID is predictive of a gene's likelihood to be linked to disease, with genes having lower Entrez Gene IDs more likely to co-occur with a disease in future publications (AUC ranging from 0.64 to 0.78). Entrez Gene IDs reflect no direct biological feature of the gene itself, but are sequentially assigned as genes are added to the database, indirectly measuring the length of time the gene has been studied. Therefore, the publication date of the oldest publication, estimating the length of publication history, and the number of publications, estimating the breadth of publication history, were examined for each gene using the Entrez Gene Feb 2007 dataset (Table 2). The AUC for the oldest publication for each gene exhibits higher predictive performance than the Entrez Gene ID number (AUC of 0.66 to 0.80), and the AUC for the number of publications is the highest of all gene-related characteristics observed (AUC of 0.73 to 0.85). Correlation of Entrez Gene ID to a richer and older publication history was reported by Leong and Kipling [35]. As the number of publications for a gene is correlated to the number of MeSH terms in the corresponding gene MeSHOP, it is not surprising that high AUC scores were obtained for MeSH term counts (Table 2; Figure 2).

**Table 2:** Comparison of the performance of Entrez Gene ID to gene-related literature measuresin MEDLINE^®^

	*gene2pubmed*			GeneRIF		
	
Feature	Validation AUC (02/2007-01/2009)	Validation AUC (02/2007-04/2010)	CTD validation (11/2008)	Validation AUC (02/2007-01/2008)	Validation AUC (02/2007-04/2010)	CTD validation (11/2008)
Number of MeSH terms	0.74	0.73	0.81	0.80	0.85	0.82
Number of publications	0.75	0.73	0.80	0.80	0.85	0.82
Oldest publication (year)	0.67	0.66	0.73	0.73	0.76	0.73
Gene ID	0.64	0.64	0.66	0.69	0.75	0.73

The oldest publication for a gene has comparable performance to Entrez Gene ID, as measured by the AUC; however, the number of publications for a gene proves to be even more predictive than the Entrez Gene ID.

As observed for gene-only score ranking, disease-only score rankings are non-random. The MeSH term counts for the disease MeSHOPs were predictive for future gene-disease co-occurrence in the literature (AUC from 0.76 to 0.90; Table 2; Figure 2). Across both gene and disease entities and across all validation sets, an entity that is highly annotated is substantially more likely to co-occur with another entity in future publications.

### MeSHOP similarity measures

Quantitative comparison of gene and disease MeSHOPs improves prediction of future gene-disease co-occurrence over the baseline values established above. Sixteen distinct similarity measures were evaluated using AUC scores, from counting measures such as term overlap and term coverage to calculated measures such as Euclidean (L_2_) and cosine distance of *P*-value profiles (Table 3). The scores evaluate the shared characteristics from both the gene and the disease MeSHOPs to make predictions. Three previously assessed baselines are presented for comparison: Entrez Gene ID, the number of terms in the gene MeSHOP, and the number of terms in the disease MeSHOP.

**Table 3:** Explanation of the scoring functions evaluated

Scoring method	Description
Cosine distance of term frequency-inverse document frequency	$\underset{j\in M}{\sum }\left(g_{i}\left(j\right)d_{i}\left(j\right)\right)/)\left(\sqrt{\underset{j\in M}{\sum }{\left(g_{i}\left(j\right)\right)}^{2}}\sqrt{\underset{j\in M}{\sum }{\left(d_{i}\left(j\right)\right)}^{2}}\right)$
Cosine distance of *P*-values	$\underset{i\in M}{\sum }\left(g_{p}\left(i\right)d_{p}\left(i\right)\right)/)\left(\sqrt{\underset{i\in M}{\sum }{\left(g_{p}\left(i\right)\right)}^{2}}\sqrt{\underset{i\in M}{\sum }{\left(d_{p}\left(i\right)\right)}^{2}}\right)$
Cosine distance of term fractions	$\underset{i\in M}{\sum }\left(g_{f}\left(i\right)d_{f}\left(i\right)\right)/)\left(\sqrt{\underset{i\in M}{\sum }{\left(g_{f}\left(i\right)\right)}^{2}}\sqrt{\underset{i\in M}{\sum }{\left(d_{f}\left(i\right)\right)}^{2}}\right)$
Sum of the log of combined *P*-values	$\underset{i\in M}{\sum }\text{log}\left(g_{p}\left(i\right)+d_{p}\left(i\right)-g_{p}\left(i\right)d_{p}\left(i\right)\right)$
Sum of the differences of log *P*-values	$\underset{i\in M}{\sum }\left|\text{log}\left(\frac{g_{p}\left(i\right)}{d_{p}\left(i\right)}\right)\right|=\underset{i\in M}{\sum }\left|\text{log}\left(g_{p}\left(i\right)\right)-\text{log}\left(d_{p}\left(i\right)\right)\right|$
L_2 _of log-p of overlapping terms only	$\sqrt{\underset{i\in \left(G\cap D\right)}{\sum }{\left(\text{log}\left(g_{p}\left(i\right)\right)-\text{log}\left(d_{p}\left(i\right)\right)\right)}^{2}}$
L_2 _of term fractions of overlapping terms only	$\sqrt{\underset{i\in \left(G\cap D\right)}{\sum }{\left(g_{f}\left(i\right)-d_{f}\left(i\right)\right)}^{2}}$
L_2 _of log of *P*-values	$\sqrt{\underset{i\in M}{\sum }{\left(\text{log}\left(\frac{g_{p}\left(i\right)}{d_{p}\left(i\right)}\right)\right)}^{2}}=\sqrt{\underset{i\in M}{\sum }{\left(\text{log}\left(g_{p}\left(i\right)\right)-\text{log}\left(d_{p}\left(i\right)\right)\right)}^{2}}$
L_2 _of *P*-values	$\sqrt{\underset{i\in M}{\sum }{\left(g_{p}\left(i\right)-d_{p}\left(i\right)\right)}^{2}}$
L_2 _of term fractions	$\sqrt{\underset{i\in M}{\sum }{\left(g_{f}\left(i\right)-d_{f}\left(i\right)\right)}^{2}}$
L_2 _of term frequency	$\sqrt{\underset{i\in M}{\sum }{\left(g\left(i\right)-d\left(i\right)\right)}^{2}}$
Term coverage	|*G*∪*D*|
Term overlap	|*G*∩*D*|
Number of gene MeSH terms	|*G*|
Number of disease MeSH terms	|*D*|
Gene ID	Entrez Gene ID of the gene

*M *refers to the set of all MeSH terms, *G *and *D *to the MeSH terms for the gene and disease profile, respectively. *g(i), g_f_(i), g_p_(i) *and *g_i_(i) *refer to the frequency, term fraction, hypergeometric *P*-value and term frequency-inverse document frequency for the MeSH term *i *of the gene profile. *d(i), d_f_(i), d_p_(i) *and *d_i_(i) *refer to the frequency, term fraction, hypergeometric *P*-value and term frequency-inverse document frequency for the MeSH term *i *of the disease profile.

The MeSHOP prediction scores produced AUC ranging from random at 0.51 to a nearly optimal AUC of 0.99, depending on the measure and the validation set (see Tables 4 and 5 for the AUC results of each score under each validation set). Each individual score was consistent across multiple validation sets and the GeneRIF or *gene2pubmed *article links, with the relative rank of the scores remaining nearly identical.

**Table 4:** Performance using GeneRIF as the gene-literature data source sets

Scoring method	Novel MEDLINE validation AUC (02/2007-01/2009)	Novel MEDLINE validation AUC (02/2007-04/2010)	Pre-existing CTD validation AUC (11/2008)	Novel CTD validation AUC (11/2008-04/2010)	Pre-existing MEDLINE validation AUC (02/2007)	Mean AUC	Rank
Cosine distance of term frequency-inverse document frequency	0.90	0.89	0.93	0.91	0.98	0.92	2
Cosine distance of *P*-values	0.56	0.57	0.60	0.56	0.53	0.56	15
Cosine distance of term fractions	0.86	0.84	0.91	0.88	0.96	0.89	4
Sum of the log of combined *P*-values	0.86	0.85	0.92	0.90	0.94	0.90	3
Sum of the differences of log *P*-values	0.91	0.91	0.77	0.83	0.93	0.87	6
L_2 _of log-p of overlapping terms only	0.94	0.93	0.91	0.92	0.98	0.94	1
L_2 _of term fractions of overlapping terms only	0.56	0.55	0.55	0.56	0.51	0.55	16
L_2 _of log of *P*-values	0.90	0.90	0.76	0.83	0.93	0.86	9
L_2 _of *P*-values	0.90	0.90	0.76	0.81	0.92	0.86	11
L_2 _of term fractions	0.86	0.85	0.89	0.88	0.94	0.88	5
L_2 _of term frequency	0.90	0.90	0.76	0.83	0.93	0.86	10
Term coverage	0.91	0.90	0.77	0.83	0.93	0.87	7
Term overlap	0.82	0.82	0.86	0.86	0.87	0.85	12
Number of gene MeSH terms	0.74	0.73	0.80	0.80	0.81	0.78	13
Number of disease MeSH terms	0.90	0.90	0.77	0.83	0.93	0.87	8
Gene ID	0.64	0.64	0.69	0.69	0.66	0.66	14

AUC of the described scoring methods were compared and tested on the validation. CTD, Comparative Toxicogenomics Database.

**Table 5:** Performance using *gene2pubmed *as the gene-literature data source

Scoring method	Novel MEDLINE validation AUC (02/2007-01/2009)	Novel MEDLINE validation AUC (02/2007-04/2010)	Pre-existing CTD validation AUC (11/2008)	Novel CTD validation AUC (11/2008-04/2010)	Pre-existing MEDLINE validation AUC (02/2007)	Mean AUC	Rank
Cosine distance of term frequency-inverse document frequency	0.92	0.91	0.95	0.93	0.98	0.94	2
Cosine distance of *P*-values	0.53	0.51	0.65	0.63	0.53	0.57	16
Cosine distance of term fractions	0.90	0.89	0.93	0.91	0.96	0.92	5
Sum of the log of combined *P*-values	0.91	0.89	0.94	0.94	0.94	0.92	3
Sum of the differences of log *P*-values	0.91	0.91	0.77	0.83	0.93	0.87	7
L_2 _of log-p of overlapping terms only	0.96	0.95	0.92	0.94	0.99	0.95	1
L_2 _of term fractions of overlapping terms only	0.64	0.62	0.57	0.60	0.53	0.59	15
L_2 _of log of *P*-values	0.90	0.90	0.76	0.83	0.93	0.86	10
L_2 _of *P*-values	0.89	0.89	0.75	0.81	0.92	0.86	12
L_2 _of term fractions	0.92	0.90	0.91	0.92	0.95	0.92	4
L_2 _of term frequency	0.90	0.90	0.76	0.82	0.93	0.86	11
Term coverage	0.90	0.91	0.77	0.83	0.93	0.87	8
Term overlap	0.91	0.89	0.90	0.92	0.90	0.90	6
Number of gene MeSH terms	0.85	0.82	0.85	0.88	0.83	0.85	13
Number of disease MeSH terms	0.90	0.90	0.76	0.83	0.93	0.86	9
Gene ID	0.75	0.73	0.78	0.79	0.74	0.76	14

AUC of the described scoring methods were compared and tested on the validation sets. CTD, Comparative Toxicogenomics Database.

Although scores such as Term Overlap and Term Coverage (mean AUC of 0.87) have high scores compared to random, these are only on par with the best baseline scores (Table 6). The most effective similarity score is the L_2 _of log-p of overlapping terms only:

**Table 6:** Summary of MeSHOP performance

Scoring method	Mean AUC	AUC standard error	Mean test rank (*n *= 200)	Overall rank
Cosine distance of term frequency-inverse document frequency	0.93	0.03	15.03	2
Cosine distance of *P*-values	0.57	0.05	87.25	16
Cosine distance of term fractions	0.90	0.04	20.21	4
Sum of the log of combined *P*-values	0.91	0.03	18.88	3
Sum of the differences of log *P*-values	0.87	0.06	26.97	7
L_2 _of log-p of overlapping terms only	0.94	0.03	12.06	1
L_2 _of term fractions of overlapping terms only	0.57	0.04	86.70	15
L_2 _of log of *P*-values	0.86	0.07	28.05	10
L_2 _of *P*-values	0.86	0.07	29.62	12
L_2 _of term fractions	0.90	0.03	20.39	5
L_2 _of term frequency	0.86	0.06	28.31	11
Term coverage	0.87	0.06	27.14	8
Term overlap	0.87	0.03	26.17	6
Number of gene MeSH terms	0.81	0.05	38.69	13
Number of disease MeSH terms	0.86	0.06	27.87	9
Gene ID	0.71	0.06	58.78	14

**Figure 3:**
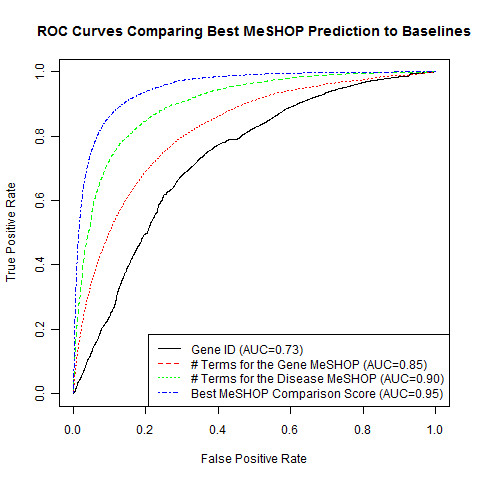
**Comparing the performance of similarity scores to gene characteristics**. ROC curves for the L_2 _of log-p of overlapping terms gene-disease profile comparison score, compared against curves for Gene ID, the number of terms in the gene MeSHOP and the number of terms in the disease MeSHOP.

The AUC mean, standard deviation and ranking for the MeSHOP scores and the gene and disease baselines are described, over all validation sets and both GeneRIF and *gene2pubmed *reference sets.

$$\sqrt{\underset{i\in \left(G\cap D\right)}{\sum }{\left(\text{log}\left(g_{p}\left(i\right)\right)-\text{log}\left(d_{p}\left(i\right)\right)\right)}^{2}}$$

where, *G *and *D *refer to the MeSH terms of gene and disease MeSHOPs, respectively, and *g_p_(i) *and *d_p_(i) *refer to the *P*-value for the MeSH term *i *of the gene or disease profile, respectively), which generates a mean AUC of 0.94 (Table 6; Figure 3). Although bibliometric baseline scores - number of article links for a gene, number of MeSH terms in the gene MeSHOP and number of terms in the disease MeSHOP - are predictive of a future paper that refers to the gene and a disease, a distinct improvement in prediction is achieved by comparing gene and disease MeSHOPs using this L_2 _score, which will be used for MeSHOP comparisons going forward.

### Alternative validation methods

As an alternative assessment to AUC scores, one can test assess a score's ability to correctly rank a list of candidate genes. For a particular disease and validation set, a list of *n *genes (for example, *n *= 200 genes) is constructed - one random disease-associated gene and *n *- 1 random non-associated genes. The list of genes is ranked by the comparison score, and the test repeated. In the case of a perfect metric, the mean test rank for the positive would be 1, and in the case of completely random predictions, the mean rank would be *n*/2. For test lists of 200 candidate genes, the top four MeSHOP comparison scores have mean test ranks from 12 to 20, nearly all ranking on average within the top 10% of the list. To compare, the mean test rank for scoring by the number of gene MeSH terms is 39 and scoring using Gene ID is 59 (Table 6).

Another alternative metric is the mean average precision (MAP). Consistent with the other metrics, the most effective MeSHOP comparison score achieves a MAP of 0.94, with the number of disease terms and the number of gene terms achieving MAP of 0.89 and 0.79, respectively (Table 7).

**Table 7:** Mean average precision MeSHOP performance

Scoring method	Novel MEDLINE validation MAP (02/2007-04/2010)	Rank	Novel CTD validation AUC (11/2008-04/2010)	Rank
Cosine distance of term frequency-inverse document frequency	0.87	11	0.92	4
Cosine distance of *P*-values	0.55	15	0.66	15
Cosine distance of term fractions	0.87	12	0.90	6
Sum of the log of combined *P*-values	0.88	9	0.94	2
Sum of the differences of log *P*-values	0.90	3	0.79	9
L_2 _of log-p of overlapping terms only	0.94	1	0.95	1
L_2 _of term fractions of overlapping terms only	0.54	16	0.52	16
L_2 _of log of *P*-values	0.89	7	0.78	13
L_2 _of *P*-values	0.89	5	0.79	8
L_2 _of term fractions	0.90	2	0.92	5
L_2 _of term frequency	0.89	8	0.79	10
Term coverage	0.90	4	0.79	11
Term overlap	0.88	10	0.93	3
Number of gene MeSH terms	0.81	13	0.88	7
Number of disease MeSH terms	0.89	6	0.78	12
Gene ID	0.69	14	0.74	14

The mean average precision for the novel MEDLINE relationships (02/2007 to 04/2010) and the novel CTD relationships (11/2008 to 04/2010). In each trial, 100 positive relationships and 100 negative relationships were chosen uniformly at random, and the average precision was computed for each scoring method. The mean average precision presented here is calculated over 100 random trials for each validation set.

### Predicting association to disease

Co-occurrence of gene and disease references in the same article does not confirm a functional relationship between the gene and the disease. Such co-occurrence could be observed for studies in which a gene-disease relationship is evaluated and found to be false or not significant, could arise from the gene and the disease appearing in separate sections, or could indicate a negative association between the gene and the disease. To address this limitation of co-occurrence analysis, the predictive capacity of MeSHOP comparison is evaluated against curated gene-disease relationships from the Comparative Toxicogenomics Database (CTD) [36,37]. Relationships for genes identified as biomarkers, therapeutic targets in treatment or playing a role in the etiology of the disease are extracted from published literature and the Online Mendelian Inheritance in Man (OMIM) database by CTD curators, and the OMIM-derived diseases are mapped onto corresponding MeSH disease terms [38]. These relationships are taken as the positive associations for ROC curve analysis to assess the MeSHOP predictions.

**Figure 4:**
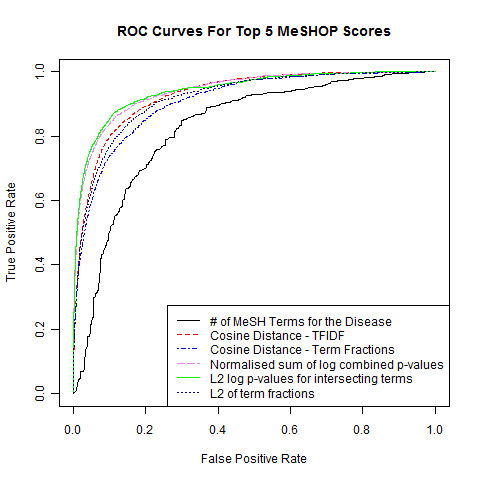
**Comparing the performance of similarity scores**. ROC curves are shown with AUC, computed for the top five similarity metrics and the disease number of MeSH terms baseline. These scores demonstrate predictions of gene-disease relationships using February 2007 data validated against the Comparative Toxicogenomics Database (11/2008) dataset.

Performance of the MeSHOP scores on the CTD validation sets are consistent with the performance seen when inferring novel disease terms for gene profiles - bibliometric baselines exhibiting up to AUC 0.85 while the best MeSHOP similarity scores achieve AUC over 0.9 (Table 2; Figures 3 and 4). Results confirm the effectiveness of MeSHOP comparison to recover *bona fide *gene-disease relationships. AUC shift by less than 0.08 when compared to the updated CTD April 2010 gene-disease relationship data (Tables 4 and 5). Similarly, when validating the prediction of new CTD relationships under the MAP metric, the strongest performing comparison score achieves MAP of 0.94, while the number of gene terms and the number of disease term bibliometric baselines achieve MAP of 0.88 and 0.78, respectively (Table 7).

### Comparative assessment of predictions with a literature-based system: candidate genes for Alzheimer disease

**Figure 5:**
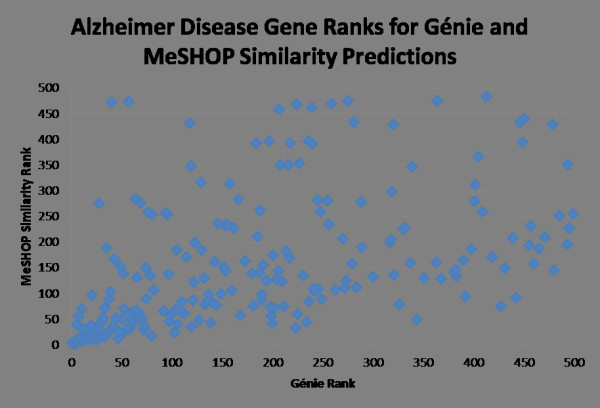
**Comparison of the top 500 gene predictions for Alzheimer disease from Génie and MeSHOP similarity**. The 215 genes ranked in the top 500 gene predictions for both Génie and MeSHOP Similarity are compared, showing a correlation of 0.38. Of the genes ranked in the top 500 by Génie, 79 did not have MeSHOPs and therefore did not have a computed MeSHOP similarity score to rank.

To place MeSHOP comparisons in relationship to a top literature-based candidate gene prediction tool, we evaluated predictions for Alzheimer disease (AD)-gene relationships by MeSHOP comparison and a leading tool. We identified the top 500 gene candidates (top 3% of genes) for AD identified by MeSHOP comparison and by the Génie system [39], plotting the relationships between the ranks in Figure 5. Génie trains a naïve linear Bayesian classifier based on the articles for the disease topic, then uses this classifier to rank the genes based on the articles associated with a gene and its homologs. The top 50 candidate genes are most strongly correlated, overlapping for 32 of the genes (see Table 8 for the top 50 candidate genes). Within Table 8, the gene candidates previously investigated in the context of AD are indicated (46 of 50 genes) with the number of articles in *gene2pubmed *for which both the gene and the AD MeSH term are attached. For Génie, 48 of the top 50 candidates co-occur with the AD MeSH term (not shown); a 49th gene - Notch3 - co-occurs with AD in two abstracts (and thus was detected as direct associations by Genie) but these papers were not curated in the *gene2pubmed *or GeneRIF sets as Notch3-focused articles. MeSHOP comparison ranked Notch3 in the top 100 candidates for AD, despite the lack of curated co-occurrence. Both systems provide highly relevant lists of genes, with MeSHOP analysis reporting more novel candidates in this particular case study. Focusing on these novel genes with no pre-existing links in the literature, the two methods both implicate the *HTT *gene, which is known to be the causative gene for the neurodegenerative disorder Huntington disease, MeSHOP comparison ranks the *XRCC3 *gene highly, a DNA repair gene that could be involved in apoptosis and neuronal cell death (both of which are mechanisms associated with AD in the literature). The most striking candidates identified may be the F2 and the F5 genes, which are involved in the blood coagulation pathway. The widely studied AD-related beta-amyloid protein has been shown to interact with fibrinogen, linking abnormalities in coagulation to the pathology of AD in recent papers [40,41].

**Table 8:** Top 50 Alzheimer disease candidate genes by MeSHOP similarity

Rank	Gene ID	Gene name	Score	Génie rank	Alzheimer disease *gene2pubmed *references
1	348	*APOE*	1.18E+04	4	812
2	351	*APP*	1.22E+04	2	576
3	4137	*MAPT*	1.23E+04	1	211
4	5663	*PSEN1*	1.27E+04	3	249
5	6622	*SNCA*	1.27E+04	6	30
6	627	*BDNF*	1.28E+04	9	47
7	1312	*COMT*	1.29E+04	87	10
8	1401	*CRP*	1.29E+04	210	5
9	6532	*SLC6A4*	1.30E+04	43	23
**10**	**3064**	**HTT**	**1.30E+04**	**44**	0
11	5444	*PON1*	1.30E+04	204	16
12	1813	*DRD2*	1.30E+04	114	1
13	4846	*NOS3*	1.30E+04	118	18
14	23621	*BACE1*	1.30E+04	5	86
15	2950	*GSTP1*	1.30E+04	470	4
16	5621	*PRNP*	1.31E+04	12	28
17	5054	*SERPINE1*	1.31E+04	NA	3
18	1636	*ACE*	1.31E+04	32	45
19	2952	*GSTT1*	1.31E+04	NA	3
20	5071	*PARK2*	1.31E+04	13	6
21	120892	*LRRK2*	1.31E+04	18	6
22	3553	*IL1B*	1.31E+04	39	32
23	4023	*LPL*	1.31E+04	172	7
24	6647	*SOD1*	1.31E+04	36	6
25	3356	*HTR2A*	1.31E+04	121	16
26	10	*NAT2*	1.31E+04	333	4
27	7515	*XRCC1*	1.31E+04	NA	2
28	2944	*GSTM1*	1.31E+04	NA	3
29	3552	*IL1A*	1.31E+04	30	36
30	3569	*IL6*	1.32E+04	60	28
31	5664	*PSEN2*	1.32E+04	7	78
32	6648	*SOD2*	1.32E+04	131	4
**33**	**2153**	**F5**	**1.32E+04**	**NA**	0
34	338	*APOB*	1.32E+04	NA	1
35	7421	*VDR*	1.32E+04	NA	2
**36**	**2147**	**F2**	**1.32E+04**	**NA**	0
37	183	*AGT*	1.32E+04	NA	2
38	1543	*CYP1A1*	1.32E+04	NA	1
39	154	*ADRB2*	1.32E+04	NA	1
40	4524	*MTHFR*	1.32E+04	57	30
41	1071	*CETP*	1.32E+04	197	8
42	3557	*IL1RN*	1.32E+04	278	7
43	4318	*MMP9*	1.32E+04	219	5
44	1565	*CYP2D6*	1.32E+04	238	9
45	335	*APOA1*	1.32E+04	135	7
**46**	**7517**	**XRCC3**	**1.32E+04**	**NA**	0
47	3990	*LIPC*	1.32E+04	NA	2
48	4153	*MBL2*	1.32E+04	NA	1
49	23435	*TARDBP*	1.32E+04	10	10
50	345	*APOC3*	1.32E+04	NA	2

Genes are ranked by MeSHOP similarity score, and compared against the ranked list of Génie candidate genes for Alzheimer disease (a full analysis considering all possible orthologs). Also provided is a list of the number of articles related to Alzheimer disease in the *gene2pubmed *references for the gene, when present. Rows in bold indicate high-ranking predictions that have no prior association with Alzheimer disease in the literature. NA: gene not among the 566 genes ranked by Génie.

### Applied analyses for prediction of gene-disease relationships

In the following sections MeSHOP gene-disease comparisons are performed for three distinct diseases: diabetes, pancreatic cancer and breast cancer. In each case, available knowledge about gene-disease relationships is identified and compared to MeSHOP comparison results. For each case, we report how many of the known genes are present in the top 500 genes (top 3%), a number selected to be suitable for manual review of results by users.

To further depict the utility of the MeSHOP comparison method, we illustrate the application of MeSHOP comparisons to predict gene-disease pairs arising in a genome-wide association study (GWAS) of diabetes. In 2007, Sladek *et al. *[42] reported a GWAS identifying eight novel risk loci for type 2 diabetes in a French cohort. Comparing the reported genes to the MeSHOP profiles (Table 9), *TCF7L2 *(Entrez Gene ID 6934) already had eight articles linking it to type 2 diabetes and hence a significant association was detected (corrected *P *= 0.018/raw *P *= 1.3e-7). As well, *IDE *(Entrez Gene ID 3416) had a weaker established link in four articles (corrected *P *= 0.50/raw *P *= 3.15e-6). No other genes emerging from the report had an established relationship to type 2 diabetes, and all six fell below the top 500 MeSHOP candidates. The gene *HHEX *(Entrez Gene 3087), which was supported by results from a subsequent diabetes study (Diabetes Genetics Initiative of Broad Institute of Harvard and MIT, 2007), was the fourth highest ranking of the eight GWAS candidates. The results indicate the potential utility for MeSHOP comparisons to aide in the interpretation of GWAS results. The absence of a high ranking MeSHOP comparison score for a relationship does not imply the relationship does not exist, but rather may reflect the limitations in the literature pertaining to the gene.

**Table 9:** Summary of diabetes loci ranked by MeSHOP similarity

Locus	Entrez Gene ID	Predicted similarity score	Rank	Percentile	Direct association
*IDE*	3416	7.59E+07	186	0.01	7.93E-02
*TCF7L2*	6934	5.91E+07	421	0.02	3.30E-03
*EXT2*	2132	2.96E+07	2616	0.10	NA
*HHEX*	3087	2.18E+07	4631	0.18	NA
*KIF11*	3832	1.87E+07	5985	0.24	NA
*ALX4*	60529	1.55E+07	8313	0.33	NA
*SLC30A8*	169026	1.55E+07	8352	0.33	NA
*LOC387761*	387761	NA	NA	NA	NA

Loci identified by (Sladek *et al. *[42]) were ranked by MeSHOP similarity (L_2 _of log-p of overlapping terms only). Direct association scores are the Bonferroni corrected *P*-values generated using the February 2007 datasets. NA

To further show that MeSHOP comparison ranks can supplement research results, we examine a study by Jones *et al. *[43] combining sequenced RNA transcripts from protein-coding genes with microarray-based detection of homozygous deletions and amplifications in pancreatic cancer. Jones *et al*. introduced a scoring procedure to differentiate causal 'driver' genes from passenger genes, identifying 6 likely driver genes within a set of 83 candidate genes (where the 83 genes were identified as having at least 2 genetic alterations in the compiled data). In Table 10, the MeSHOP comparison scores for 'pancreatic neoplasms' are reported for the 83 identified genes. Five of the six Jones *et al*.-ranked driver candidate genes (*TP53, CDKN2A, KRAS, TGFBR2, SMAD4*) were represented in the top 500 MeSHOP predictions; MeSHOP comparisons did not identify the sixth gene, *MLL3*. The *EP300 *gene was low scoring in the Jones *et al*. rankings, although it was ranked fifth overall by MeSHOP comparisons. *EP300 *has been shown to be downregulated by microRNA in highly metastatic pancreatic ductal adenocarcinomas [44], demonstrating the capacity for MeSHOP comparison to identify candidate disease genes. The MeSHOP comparison provides a bibliometric view that both reinforces and complements existing analytical methods.

**Table 10:** Comparison of MeSHOP results for pancreatic cancer candidate genes

Gene	Entrez gene	Predicted similarity	Rank	Percentile	Mutations	Deletions	Passenger probability: low rates	Passenger probability: mid rates	Passenger probability: high rates
*TP53*	7157	1.24E+08	11	100	18	2	< 0.001	< 0.001	< 0.001
*CDKN2A*	1029	8.29E+07	135	99	2	16	< 0.001	< 0.001	< 0.001
*KRAS*	3845	6.95E+07	266	99	24	0	< 0.001	< 0.001	< 0.001
*TGFBR2*	7048	6.76E+07	288	99	3	1	< 0.001	0.001	0.003
*EP300*	2033	6.37E+07	351	99	2	0	0.176	0.482	0.984
*SMAD4*	4089	6.14E+07	386	98	8	6	< 0.001	< 0.001	< 0.001
*ELN*	2006	5.57E+07	509	98	2	0	0.115	0.372	0.413
*F8*	2157	5.51E+07	530	98	2	0	0.165	0.482	0.853
*SCN5A*	6331	5.18E+07	629	98	2	0	0.176	0.482	1.000
*PRKCG*	5582	4.77E+07	798	97	2	0	0.115	0.372	0.413
*TPO*	7173	4.71E+07	831	97	2	0	0.115	0.375	0.694
*PPP1R3A*	5506	4.50E+07	946	96	2	0	0.115	0.477	0.694
*SMARCA4*	6597	4.04E+07	1243	95	2	0	0.062	0.183	0.413
*COL5A1*	1289	3.72E+07	1518	94	2	0	0.176	0.482	0.984
*MEP1A*	4224	3.38E+07	1895	92	2	0	0.062	0.183	0.413
*IL2RG*	3561	2.95E+07	2652	89	1	0	0.004	0.016	0.997
*ATP10A*	57194	2.77E+07	2974	88	2	0	0.176	0.482	1.000
*MYH2*	4620	2.71E+07	3063	88	2	0	0.165	0.477	0.853
*GRIA3*	2892	2.62E+07	3281	87	1	1	0.017	0.069	0.999
*ABCA7*	10347	2.56E+07	3426	86	2	0	0.033	0.139	0.201
*DLG3*	1741	2.51E+07	3540	86	1	0	0.003	0.015	0.997
*DLC1*	10395	2.47E+07	3645	86	2	0	0.176	0.482	1.000
*GLTSCR1*	29998	2.06E+07	5082	80	2	0	0.062	0.183	0.405
*PCSK6*	5046	2.02E+07	5240	79	2	0	0.176	0.482	0.911
*EVPL*	2125	2.00E+07	5329	79	2	0	0.176	0.482	0.942
*NRG2*	9542	1.95E+07	5537	78	2	0	0.165	0.477	0.853
*SLITRK5*	26050	1.93E+07	5655	78	2	0	0.165	0.477	0.853
*SEMA5B*	54437	1.92E+07	5713	77	2	0	0.062	0.183	0.413
*DPP6*	1804	1.86E+07	6025	76	3	0	0.009	0.079	0.201
*PCDH15*	65217	1.84E+07	6162	76	4	0	< 0.001	0.017	0.048
*FMN2*	56776	1.82E+07	6266	75	2	0	0.176	0.482	0.911
*CACNA2D1*	781	1.77E+07	6597	74	1	0	0.001	0.004	0.989
*DLEC1*	9940	1.70E+07	7039	72	2	0	0.176	0.482	0.911
*MLL3*	58508	1.69E+07	7090	72	6	0	< 0.001	< 0.001	< 0.001
*PB1*	55193	1.63E+07	7597	70	2	0	0.165	0.477	0.853
*LRRN3*	54674	1.60E+07	7856	69	2	0	0.062	0.183	0.405
*CYFIP1*	23191	1.56E+07	8225	67	3	0	0.009	0.079	0.201
*SF3B1*	23451	1.55E+07	8290	67	3	0	0.009	0.079	0.201
*PXDN*	7837	1.55E+07	8302	67	2	0	0.176	0.482	1.000
*TNR*	7143	1.54E+07	8453	66	2	0	0.176	0.482	0.911
*SN*	6614	1.53E+07	8484	66	2	0	0.176	0.482	1.000
*SLC6A15*	55117	1.53E+07	8488	66	2	0	0.062	0.183	0.405
*ARID1A*	8289	1.51E+07	8688	66	2	0	0.176	0.482	0.984
*SLC1A6*	6511	1.48E+07	8908	65	2	0	0.115	0.477	0.694
*LRRTM4*	80059	1.46E+07	9064	64	2	0	0.062	0.183	0.413
*GALNT13*	114805	1.42E+07	9651	62	2	0	0.062	0.183	0.405
*GUCY1A2*	2977	1.39E+07	9964	60	2	0	0.062	0.183	0.405
*ZNF638*	27332	1.37E+07	10174	60	2	0	0.115	0.375	0.694
*PDZRN3*	23024	1.33E+07	10522	58	2	0	0.033	0.082	0.201
*DOCK2*	1794	1.33E+07	10612	58	2	0	0.062	0.183	0.405
*MIZF*	25988	1.32E+07	10714	58	2	0	0.062	0.183	0.405
*DACH2*	117154	1.30E+07	10883	57	1	1	0.022	0.088	1.000
*ST6GAL2*	84620	1.26E+07	11302	55	2	0	0.115	0.375	0.694
*KBTBD11*	9920	1.19E+07	12083	52	1	1	0.006	0.025	0.998
*CNTN5*	53942	1.18E+07	12231	51	2	0	0.115	0.375	0.694
*ABLIM2*	84448	1.17E+07	12471	51	2	0	0.062	0.183	0.405
*PCDH18*	54510	1.14E+07	12864	49	2	0	0.115	0.375	0.694
*ADAMTS20*	80070	1.09E+07	13668	46	2	0	0.176	0.482	0.911
*CDH10*	1008	1.09E+07	13703	46	3	0	< 0.001	0.017	0.048
*KIAA1024*	23251	1.09E+07	13715	46	2	0	0.115	0.375	0.694
*TBX18*	9096	1.08E+07	13821	45	2	0	0.062	0.183	0.413
*LRFN5*	145581	1.07E+07	13894	45	2	0	0.062	0.183	0.405
*DEPDC2*	80243	1.07E+07	13953	45	3	0	0.055	0.183	0.405
*FMNL3*	91010	1.05E+07	14376	43	2	0	0.055	0.179	0.405
*TM7SF4*	81501	1.03E+07	14681	42	2	0	0.055	0.179	0.405
*OR10R2*	343406	1.02E+07	15126	40	2	0	0.033	0.139	0.317
*GPR133*	283383	1.02E+07	15188	40	2	0	0.062	0.183	0.405
*PCDH17*	27253	1.01E+07	15355	39	2	0	0.062	0.183	0.405
*BAI3*	577	9.58E+06	16457	35	3	0	0.033	0.082	0.201
*KIAA0774*	23281	9.49E+06	16660	34	2	0	0.176	0.482	0.984
*CTNNA2*	1496	9.42E+06	16781	33	3	0	0.033	0.179	0.405
*KLHDC4*	54758	8.66E+06	18571	26	2	0	0.033	0.082	0.201
*ZAN*	7455	8.45E+06	19030	25	2	0	0.176	0.482	0.984
*DKFZP586P0123*	26005	7.38E+06	20579	18	2	0	0.165	0.477	0.853
*UNC13C*	440279	7.38E+06	20835	17	2	0	0.115	0.372	0.694
*FLJ39155*	133584	7.38E+06	21333	15	2	0	0.176	0.482	0.942
*RASSF6*	166824	7.38E+06	21543	15	2	0	0.062	0.183	0.405
*OVCH1*	341350	5.79E+06	24923	1	2	0	0.165	0.477	0.853
*Q9H5F0_HUMAN*		NA	NA	NA	3	0	< 0.001	0.004	0.009
*Q9H8A7_HUMAN*		NA	NA	NA	2	0	0.165	0.477	0.853
*FLJ46481*	389197	NA	NA	NA	2	0	0.062	0.183	0.405
*XR_017918.1*		NA	NA	NA	2	0	0.062	0.183	0.405
*LOC441136*	441136	NA	NA	NA	2	0	0.009	0.079	0.201

This table shows all genes from Supplementary Table S7 of Jones *et al. *[43] listed by strength of MeSHOP similarity score (via the L_2 _of log-p of overlapping terms only metric). NA, no MeSHOP available.

A recent publication examined the genomes of 100 breast cancer tumors for somatic mutations looking for potential driver mutations [45], reporting 13 somatic cancer genes with seven or more observed mutations. Six of the thirteen reported genes are in the top thirteen MeSHOP candidates (Table 11). Within the top 500 MeSHOP candidates, including the set of 6, a total of 11 of the 13 somatic cancer genes were present. Top MeSHOP candidates that were not reported in the somatic breast cancer 'driver' gene list include several genes with known hereditary roles in breast cancer (for example, *BRCA1 *(MeSHOP rank 4) and *BRCA2 *(MeSHOP rank 8); Table 12).

**Table 11:** MeSHOP similarity analysis of known breast cancer genes with seven or more observed mutations

Chromosomal location/gene(s)	Mutations observed	MeSHOP similarity rank
*TP53*	*38*	*1*
PIK3CA	33	55
*chr17:37833600-38018803/ERBB2*	*21*	*2*
GATA3	15	773
chr8:37353781-37489508/FGFR1/ZNF703	15	191/8,493
chr8:128504497-128948225/MYC	15	19
*chr11:69224506-69556470/CCND1*	*11*	*9*
MAP3K1	9	417
chr20:52065876-52723895/ZNF217	9	2,102
*PTEN*	*7*	*12*
NCOR1	7	360
*CDKN2A*	*7*	*6*
*CDH1*	*7*	*11*

Genes known to be implicated in breast cancer from Supplementary Table 4 of [45] are compared to the MeSHOP similarity ranking of human genes for breast neoplasms. The rows in italics highlight genes with a MeSHOP similarity rank less than 13.

**Table 12:** Highest ranked breast cancer gene candidates with MeSHOP similarity analysis

Rank	Gene	Probability of oncogenicity	MeSHOP similarity rank
**1**	MAP3K1	**1**	**417**
2	TBX3	0.99996	2513
3	TTN	0.99952	1842
**4**	NCOR1	**0.99835**	**360**
5	MTMR4	0.98201	10529
6	MAP3K13	0.97383	9545
**7**	CDKN1B	**0.96378**	**22**
8	DIDO1	0.90433	4931
9	SMARCD1	0.88007	3098
**10**	CASP8	**0.86462**	**150**

We list the top ten breast cancer candidate genes from Supplementary Table 5 of [45], ranked by probability of oncogenicity. Genes in bold are in the top 500 by MeSHOP similarity rank.

Together these case studies demonstrate the utility of MeSHOP predictions as a complement to laboratory studies, providing support to a subset of candidate genes, revealing classes of genes not detected, and highlighting novel genes for further investigation.

### Availability of results

Results are freely accessible on the web [46]. MeSHOP profiles can be browsed online for genes [47] and for diseases [48]. Gene-disease MeSHOP profile-based predictions can also be viewed online [49], listing results by disease (listing the most similar genes), or by gene (listing the most similar diseases), sorted by similarity score. Specific datasets relevant to this paper can be directly downloaded [50].

## Discussion

Quantitative annotation profiles based on MeSH annotations, MeSHOPs, are shown to facilitate the identification of gene-disease relationships. In assessing the baseline properties of gene-disease relationship predictions, we observe a striking bias introduced by the level of annotation of the entities (gene and/or disease), such that simply predicting future gene-disease relationships based on the most studied genes (or diseases) is better than random guessing. Accounting for this bias, we demonstrate that comparison metrics using MeSHOPs has high capacity to predict future gene-disease co-occurrence in future research publications. Functional relationships between genes and diseases were predicted using reference collections, and shown to perform better than baselines. Overall, MeSHOP comparison is shown to be a useful tool for applied bioinformatics.

Strong performance of bibliometric baselines quantitatively indicates researchers may tend to explore additional relationships for existing well-characterized genes and diseases, echoing the imbalanced research activity seen by Agarwal and Searls [51]. On the other hand, this may rather reflect methodological biases emphasizing easier to characterize genes and diseases. Well-studied genes have pre-existing protocols and materials such as animal models and PCR primers. Well-studied diseases may be more commonly and reliably identified through better-established diagnostic methods and physician familiarity. Rather than bias, it may reflect properties of a subset of genes and diseases. Certain types of genes and diseases are involved in key processes, similar to multifunctional proteins in interaction networks [52]. A 'hub' gene involved in many pathways could cause many phenotypes when disrupted. Similarly, some phenotypes may actually be the result of many different molecular processes, each of which when misregulated due to a gene can cause different variations of the disease phenotype. As well, there are not just the causative genes for a disease, but many other genes may regulate the severity or provide protective immunity against the disease phenotype. Regardless of origin of these observed predictive biases, we strongly recommend that all future gene-disease prediction methods be contrasted to gene and disease bibliometric baseline characteristics - ideally against the strongest metrics that evaluate the degree of annotation (the number of MeSH terms in the MeSHOPs for the gene and disease). Bibliometric baseline comparison allows direct comparative assessment of the predictive ability of methods compared to these universal trends.

Previous work demonstrated gene length, cDNA length and protein length significantly differ between control and disease genes [53,54]. Our literature-based analysis shows neither genomic length nor transcript length have significant predictive ability in our current validation sets, suggesting these previous biases are no longer foretelling of future gene-disease association. Advances in methodology such as high-resolution microarrays and sequencing may have removed the influence of the bias, suggesting that literature bias favoring well-studied genes may correct itself as more genes become better characterized.

The L_2 _of log-p of overlapping terms similarity metric outperformed all other methods, but many methods performed nearly as well. The highest-performing metrics all focus on the terms shared by the profiles and emphasize terms unlikely to be associated by chance. The L_2 _metric is conceptually straightforward and supported by all statistical and mathematical analysis packages. The fact that multiple metrics perform well suggests that the performance may be constrained by the data properties. The quality of MeSH annotations appears high, as MeSH annotations are assigned by domain experts at the NLM. However, the limited time that can be devoted to any single article necessarily means that the assigned terms are limited to the main topics of the articles. MeSHOPs and comparison methods may benefit from more comprehensive annotation assignments based on automated full-text analysis.

### Comparison to other MeSH-related methods

A related but different method of CoPub Discovery [55] seeks to identify hidden links between genes and diseases through shared keywords in MEDLINE^® ^abstracts. They assess predictions using historical entries from before 2000, identifying genes and keywords from entries using text mining. As a comparison scores, they employ a straightforward linear summation of the minimum score of the shared MeSH terms. In contrast, our predictions use the larger corpus of MEDLINE^® ^up to 2007, and our MeSHOPs-based method builds on curated MeSH terms and Entrez Gene article links, enabling a broad range of applications. We also evaluate measures of MeSH term association strength to generate MeSHOPs and many different comparison scores for comparing gene and disease MeSHOPs.

The text mining method reported by Srinivasan [56] extracts MeSH terms of importance to summarize a set of articles related to an entity. This considers common MeSH terms between profiles as potential paths to connect two entities. MeSHOPs use a statistical scoring method to compute *P*-values for the profiles, and further evaluates a large number of different methods for generating and comparing the MeSHOPs, analyzing all terms between profiles computationally.

Sarkar *et al. *[14] use weighted profiles of MeSH terms and visualize the terms as a MeSH cloud to summarize a collection of documents retrieved from MEDLINE^® ^and to facilitate further investigation of related articles in MEDLINE^®^.

MeSHOPs share conceptual similarities with the method of CAESAR [22]. CAESAR scores the occurrences of extracted keyword terms in an authoritative text that summarizes the topic of interest. MeSHOPs use all relevant articles, each with individual associated MeSH biomedical terms, reflecting both the main directions of research and associated topics.

### Future directions

The use of MeSHOPs to infer term attachment and predict novel associations need not necessarily be limited to the attachment of disease terms to genes and vice versa. This methodology could be expanded to the attachment of any subset of MeSH terms to biomedical topics of interest.

MeSHOPs could be explored for gene-disease associations in other species than human - preliminary analysis predicting mouse genes associated with MeSH disease terms have achieved similar performance results. Human disease gene prioritization has been shown to be improved through incorporation of mouse phenotype data [25], suggesting incorporation of orthology data could be used to improve predictions. New candidates for complex diseases could also be evaluated through their similarity to known genes related to the disease of interest, as seen in an analysis by Taniya *et al. *[57] for rheumatoid arthritis and prostate cancer using several other sources of gene annotation.

## Conclusions

MeSHOPs quantitatively represent the MeSH biomedical terms associated with any defined entity with an identified set of MEDLINE^®^-indexed papers. Results demonstrate MeSHOP similarity can infer functional annotation of genes and diseases. Specifically, the similarity between gene MeSHOPs and disease MeSHOPs is highly predictive of future gene-disease ties. Although bibliometric characteristics, such as the number of terms in the disease MeSHOP, are predictive of gene-disease association, our best predictions, using L_2 _of log-p of overlapping terms, achieve a mean AUC of 0.94, an improvement of 0.08 over the mean AUC for the number of disease terms baseline, and an improvement of 0.13 over the mean AUC for the number of gene terms baseline. The consistency of the results over five validation sets duplicated over two sources of gene article links demonstrates that the predictive performance of our method is stable and replicable. Beyond the prediction of annotation, MeSHOP comparison predicts genes with functional roles in disease process, validated using curated gene-disease relationships in CTD and in case studies.

## Materials and methods

### MeSHOP generation for genes and diseases

We describe here the construction of MeSHOPs - a detailed introduction and establishment of this method is presented elsewhere [31]. A MeSHOP is a quantitative representation of the MeSH annotations associated with a set of articles where the set is composed of articles that address a specific entity (such as a gene or disease). The computation of a MeSHOP initiates from a set of articles that address a specific entity. Each article has a curator-assigned set of MeSH terms available in MEDLINE^®^. Comparing the observed frequency of each MeSH term annotated to a set of articles relative to the background rate for each term returns a measure of over-representation. A MeSHOP is a vector of tuples < (*t_1_, m_1_*), (*t_2_, m_2_*), ... (*t_n_, m_n_*) >. For each tuple (*t_i_, m_i_*) in a MeSHOP, *t_i _*is a distinct MeSH term in the MeSH vocabulary and *m_i _*is the numeric measure of the strength of association of the MeSH term *t_i _*to the set of articles (for example, the over-representation measures). To account for the tree structure of MeSH, for each MeSH term associated with an article, the article is considered associated to all of the parent terms of that MeSH term.

Several scoring metrics have been implemented to report the strength of association between an entity and a MeSH term in a quantitative fashion. A basic measure is the raw count of articles annotated with each term. Such counts can be normalized by dividing the raw count by the total number term annotations for the particular gene or disease to address the degree of annotation. However, counting methods fail to account for statistical significance; the frequency in which term appears in MEDLINE^® ^should be accounted for. To address this deficiency, *P*-values can be computed based on a hypergeometric distribution via Fisher's exact test.

MeSHOPs are generated for each member of a class by assessing the set of all linked MEDLINE^® ^records for each member. We use Fisher's exact test to determine *P*-values, computed from a 2 × 2 contingency table composed of: 1) the frequency of occurrence of the term *t_i _*in the set of articles addressing the entity of interest; 2) all articles addressing the entity of interest without the term *t_i_*; 3) the frequency of the term *t_i _*in the background set not addressing the entity of interest; and 4) the remaining number of articles in the background set that do not refer to the term *t_i _*and do not address the entity of interest. A universal background of MeSH term frequency is applied in this case derived from a set of 17 million MEDLINE^® ^articles assigned MeSH terms. For every MeSH term that occurs in the articles associated with a gene, we compute the statistical significance of the association. Entity class-specific MeSH frequency backgrounds can be applied for improved visualization when comparisons are not sought as described in [31]; in this paper, however, we use the universal background described here that is common to both genes and diseases.

### Inferring novel gene-disease association

To infer entity-MeSH annotation relationships, we hypothesize that a previously unassociated MeSH term *t *is likely to be associated with an entity *e *if the MeSHOP *P_t _*for the MeSH term *t *is highly similar to the entity's MeSHOP *P_e_*. The scoring of similarity was performed with a panel of formulae presented in Table 3. Once the profiles for each human gene and each disease term in MeSH were computed, we measured the similarity of each human gene profile against each disease profile.

### Quantitative validation of gene-disease similarity measures

ROC curves were computed for each of the similarity scores evaluated. AUC was measured to assess the accuracy of the scoring metrics. In the case where there are no ties, the ROC curve is composed of horizontal and vertical sections; in the case of ties, diagonal sections also occur. AUC values can be converted to mean rankings by noting that the AUC reports the mean probability that, for a random disease, given a random positive gene and a random negative gene, the positive gene is scored higher than the negative gene. The ranking of the positive is the result of *n *- 1 Bernoulli trials, where the positive is compared to each of the negatives. Each 'failure' in this case causes the rank to drop by 1. The average rank is given by 1 + (1 - AUC)(*n *- 1).

### Data sources

The annual MEDLINE^® ^Baseline releases 2007, 2009 and 2010 were used as the source of MeSH annotations for articles. All gene-disease co-occurrences (that is, the gene and the disease directly linked to the same article) were extracted for each release. Similarly, we manually downloaded snapshots of Entrez Gene, including the links from genes to MEDLINE^®^/PubMed^® ^articles from GeneRIF and *gene2pubmed*, approximately matched to the date of the MEDLINE^® ^releases. For each MEDLINE^® ^Baseline release matched with Entrez Gene downloaded snapshot, we generated the MeSHOP for each disease in MeSH, the MeSHOP for each human gene using the associated GeneRIF annotation, and the MeSHOP for each human gene using the associated *gene2pubmed *annotation. We compared each gene MeSHOP with GeneRIF annotation against each disease using the similarity scores. Each gene MeSHOP with *gene2pubmed *annotation was also compared against each disease using the similarity scores. See Table 13 for details of the size and contents of these datasets. We use the references from the 2007 Entrez Gene snapshot and the 2007 MEDLINE^® ^Baseline to generate MeSHOP similarity scores for all human genes with GeneRIF annotations. The MeSHOP similarity scores for all human genes with *gene2pubmed *annotations were also generated. These two sets of gene-disease MeSHOP similarity scores were validated for the ability to predict novel co-occurrences of genes and diseases in MEDLINE^®^, as well as the ability to predict new curated gene-disease relationships.

**Table 13:** Datasets used in the analysis with details on size and relevant contents

Dataset	February 2007	January 2009	April 2010
Entrez Gene (including *gene2pubmed *and GeneRIF)			
Total genes	2,460,748	4,710,910	5,999,558
Human genes	38,604	40,183	45,423
			
	Baseline 2007 (Nov 2006)	Baseline 2009 (Nov 2008)	Baseline 2010 (Nov 2009)
MEDLINE^®^			
Total articles	16,120,073	17,764,232	18,502,915
			
*gene2pubmed *(Linking Entrez Gene and MEDLINE^®^			
Total links	3,081,413	12,960,489	5,979,167
Total human gene links	272,123	445,650	527,821

Although the number of human genes has not increased much over the years, the number of non-human links has increased substantially since 2007, while the human gene links have increased at a more moderate rate. Previously, MEDLINE^®^/PubMed^® ^links from genomic sequence were propagated to all related genes. This practice was discontinued in March 2009, resulting (at the time) in a 60% decrease in links and the disparity in the number of overall links from 2009 to 2010.

### Gene-disease novel co-occurrence validation sets

To validate the effectiveness of predicting using MeSHOP similarity, we generated predictions using archived versions of all the datasets (MEDLINE^® ^and Entrez Gene), involving data up to 2007. Using more recent versions of MEDLINE^® ^and Entrez Gene, we identify new gene-disease relationships that appeared after 2007. These novel relationships are considered the true positives for validation. We then evaluated the accuracy of the MeSHOP similarity comparison of the 2007 data at predicting novel gene-disease relationships appearing after 2007, quantitatively evaluated using ROC AUC. We also measured the accuracy of the similarity measures at predicting the existing gene-disease relationships up to 2007.

Gene characteristics were extracted from EnsEMBL 53 (April 2009) and these characteristics were mapped to the human genes in Entrez Gene. The genes with mapped gene characteristics were then evaluated for the ability to predict novel gene-disease predictions, providing a baseline to contrast the performance of the MeSHOP similarity predictions.

### Novel curated gene-disease relationship validation sets

Two sets of curated gene-disease relationships were extracted from the CTD. These gene-disease relationships are identified from OMIM and published MEDLINE^® ^literature by CTD curators. The first set was all gene-disease tuples involving MeSH disease terms downloaded November 2008 (3,630 tuples covering 828 diseases and 2,092 genes for the genes with *gene2pubmed*-based MeSHOPs, and 3,178 tuples covering 780 diseases and 1,714 genes for the genes with GeneRIF-based MeSHOPs). We compared these relationships with the relationships present in the version of CTD downloaded April 2010, and created a validation set where the 'true positives' composed of all the new tuples present in the April 2010 dataset that were not present in the November 2008 dataset (1,760 new tuples covering 426 diseases and 1,068 genes for the genes with *gene2pubmed*-based MeSHOPs, and 1,602 new tuples covering 409 diseases and 942 genes for the genes with GeneRIF-based MeSHOPs). This validation set was used to evaluate the performance of the MeSHOP similarity scores generated using MEDLINE^® ^and Entrez Gene data up to 2007. Gene characteristic baselines were also evaluated to contrast the performance of the MeSHOP similarity scores.

### Predictive performance for pre-existing relationships

To confirm the expected capacity for MeSHOP comparisons to detect established gene-disease relationships, we evaluated the performance of similarity measures at ranking pre-existing gene-disease relationships (relationships reported in the literature before 2008). This assessment was performed for gene-disease co-occurrences obtained from Entrez Gene and MEDLINE^® ^as well as from the November 2008 relationships curated from CTD.

### Examining overlapping terms

While MeSHOP similarity scores provide a quantitative assessment of the similarity of a gene MeSHOP and a disease MeSHOP, examining the overlapping terms between the profiles and the associated *P*-values could also provide insight into the medical topic areas that are shared by the MeSHOPs and provide clues into how the concepts can be related. We provide a link when browsing the MeSHOP similarity scores online to the list of overlapping MeSH terms between the two profiles, ordered by the similarity of the *P*-values for the shared terms. An example showing the top 100 shared terms between *PAX6 *and anirida is provided in Table 14, showing both general terms relating to genetics and heritable diseases such as 'mutation, missense' and 'exons', as well as terms specifically linking the gene to disease such as 'chromosomes, human, pair 11' and 'cataract'.

**Table 14:** Top 100 terms shared by the MeSHOPs of *PAX*6 and aniridia

Common MeSH term	Gene MeSHOP *P*-value	Disease MeSHOP *P*-value	Score
DNA mutational analysis	0.00E+00	0.00E+00	0.00e+0
Pedigree	0.00E+00	0.00E+00	0.00e+0
Polymorphism, single-stranded conformational	1.40E-44	1.67E-42	1.66e-42
Humans	6.82E-24	0.00E+00	6.82e-24
Exons	8.53E-24	2.98E-23	2.13e-23
Mutation, missense	1.45E-23	9.72E-21	9.70e-21
Chromosomes, human, pair 11	2.73E-20	0.00E+00	2.73e-20
Codon, nonsense	1.15E-18	1.96E-21	1.15e-18
Cataract	2.37E-17	0.00E+00	2.37e-17
Point mutation	6.94E-17	7.02E-18	6.24e-17
Frameshift mutation	9.77E-15	2.11E-21	9.77e-15
DNA primers	5.27E-12	2.69E-15	5.27e-12
Fovea centralis	2.41E-16	6.03E-11	6.03e-11
Introns	5.01E-10	2.15E-13	5.01e-10
Nystagmus, congenital	9.55E-10	3.29E-11	9.22e-10
Genes, dominant	7.39E-09	2.45E-14	7.39e-9
Asian continental ancestry group	2.23E-16	1.07E-08	1.07e-8
Lens, crystalline	2.40E-08	5.62E-24	2.40e-8
Alternative splicing	6.14E-13	7.97E-08	7.97e-8
Corneal opacity	2.45E-06	1.47E-16	2.45e-6
Child, preschool	3.63E-06	3.08E-44	3.63e-6
Family health	1.03E-05	1.69E-07	1.01e-5
Gene expression regulation, developmental	2.46E-15	1.04E-05	1.04e-5
Genes, homeobox	1.40E-05	6.32E-09	1.40e-5
Adolescent	1.92E-05	1.71E-18	1.92e-5
Conserved sequence	6.20E-06	1.18E-04	1.12e-4
Heterozygote	1.15E-04	6.94E-08	1.15e-4
Radiation hybrid mapping	1.72E-05	1.50E-04	1.33e-4
Alleles	2.29E-04	4.17E-05	1.87e-4
Abnormalities, multiple	2.91E-04	0.00E+00	2.91e-4
Iris	3.45E-04	0.00E+00	3.45e-4
Blepharoptosis	4.54E-04	4.55E-08	4.53e-4
WAGR syndrome	5.13E-04	0.00E+00	5.13e-4
Tomography, optical coherence	1.14E-03	4.47E-04	6.97e-4
Corpus callosum	6.03E-07	9.38E-04	9.38e-4
Pregnancy	9.62E-01	9.60E-01	1.09e-3
Open reading frames	2.56E-10	1.12E-03	1.12e-3
Forkhead transcription factors	1.27E-03	1.95E-05	1.25e-3
Face	1.42E-03	3.94E-05	1.38e-3
Nucleic acid heteroduplexes	2.02E-04	1.73E-03	1.53e-3
*In situ *hybridization, fluorescence	1.61E-03	3.22E-29	1.61e-3
Gene deletion	1.65E-03	1.77E-21	1.65e-3
*PAX9 *transcription factor	8.46E-04	2.50E-03	1.66e-3
Proprotein convertase 1	1.71E-03	1.27E-05	1.69e-3
Ectopia lentis	1.81E-03	1.42E-05	1.79e-3
Albinism, ocular	1.86E-03	3.98E-14	1.86e-3
Databases, nucleic acid	3.09E-04	2.63E-03	2.32e-3
India	1.13E-04	2.79E-03	2.68e-3
Amino acid substitution	2.03E-06	3.07E-03	3.06e-3
Transcriptional activation	1.10E-23	3.22E-03	3.22e-3
Genetic markers	3.43E-03	1.71E-10	3.43e-3
Anophthalmos	5.77E-03	1.45E-04	5.63e-3
3' Untranslated regions	8.26E-06	5.66E-03	5.65e-3
Young adult	1.18E-02	4.91E-03	6.84e-3
Limbus corneae	7.58E-03	1.48E-18	7.58e-3
RNA, transfer, Lys	4.19E-03	1.23E-02	8.16e-3
Dna transposable elements	9.49E-03	9.94E-04	8.50e-3
Heteroduplex analysis	4.54E-03	1.34E-02	8.84e-3
Chromosome deletion	9.12E-03	0.00E+00	9.12e-3
Homozygote	1.09E-02	1.29E-03	9.57e-3
Otx transcription factors	5.70E-06	1.00E-02	9.99e-3
Genetic predisposition to disease	1.16E-02	8.84E-05	1.15e-2
Microphthalmos	1.17E-02	2.34E-12	1.17e-2
Vision, low	1.24E-02	6.65E-04	1.17e-2
Optic nerve	1.26E-02	5.64E-08	1.26e-2
Exotropia	7.21E-03	2.12E-02	1.40e-2
Cytosine	2.70E-03	2.15E-02	1.88e-2
Magnetic resonance imaging	4.92E-04	1.96E-02	1.92e-2
United States	9.81E-01	1.00E+00	1.93e-2
Trabecular meshwork	2.22E-02	2.11E-03	2.01e-2
Polymorphism, restriction fragment length	2.30E-02	7.12E-04	2.23e-2
Body patterning	3.32E-03	2.62E-02	2.29e-2
Dichotic listening tests	1.21E-02	3.55E-02	2.34e-2
Multigene family	2.46E-02	8.37E-04	2.38e-2
3T3 Cells	3.00E-06	2.67E-02	2.67e-2
Cognition disorders	4.77E-02	2.05E-02	2.72e-2
Esotropia	1.42E-02	4.14E-02	2.72e-2
Mutagenesis, insertional	4.08E-03	3.18E-02	2.77e-2
Endothelium, corneal	2.94E-02	1.06E-04	2.93e-2
Restriction mapping	3.25E-02	2.53E-06	3.25e-2
Thymine	4.15E-02	7.24E-03	3.43e-2
Sequence homology, amino acid	3.52E-02	3.31E-04	3.49e-2
Glutamine	5.38E-03	4.13E-02	3.59e-2
Chromosomes, human, pair 10	1.97E-02	5.72E-02	3.75e-2
Cytogenetics	3.86E-02	2.41E-04	3.84e-2
Nervous system malformations	5.11E-02	9.30E-02	4.18e-2
Organ specificity	2.36E-01	1.90E-01	4.62e-2
Catenins	6.22E-02	1.59E-02	4.63e-2
Genetic heterogeneity	2.45E-02	7.09E-02	4.64e-2
Brain-derived neurotrophic factor	5.07E-02	6.24E-07	5.07e-2
Chromosomes, human, pair 12	2.70E-02	7.77E-02	5.08e-2
Leucine zippers	1.64E-04	5.28E-02	5.26e-2
Verbal behavior	2.09E-01	1.53E-01	5.57e-2
Mice, transgenic	6.20E-02	5.89E-03	5.61e-2
Visual acuity	5.65E-02	0.00E+00	5.65e-2
DNA fingerprinting	9.30E-02	3.44E-02	5.85e-2
Sequence alignment	1.69E-02	7.59E-02	5.90e-2
Autistic disorder	9.48E-02	3.57E-02	5.91e-2
Fluorescent antibody technique, indirect	9.84E-02	3.83E-02	6.00e-2
Age factors	9.37E-01	9.97E-01	6.03e-2

The top 50 most similar MeSH terms of the 235 MeSH terms shared by both the MeSHOP for aniridia and the MeSHOP for *PAX6 *are presented here. The *P*-value of the term in the gene MeSHOP and the disease MeSHOP are presented, and ordered by the difference in the two *P*-values.

### Implementation

The analysis was performed using Python 2.5.2 [58], XSLT [59], and the MySQL database system 5.0.51a [60]. Fisher's exact test *P*-values were computed using the R statistics package [61]. Results were generated using 50 CPUs of a compute cluster running under Grid Engine 6.2 [62]. A typical cluster machine is a 64-bit dual processor 3 GHz Intel Xeon with 16 GB of RAM.

Datasets were downloaded from Entrez Gene [63] - including the *gene2pubmed *and GeneRIF links - and MEDLINE^® ^via a lease from the NLM [64]. The CTD validation set was taken from the gene-disease relationships dataset [65].

### Availability of source code

Source code is available for the gene and disease profile analysis [66], and for the evaluation and validation of results [67].

## Abbreviations

AD: Alzheimer disease; AUC: area under the ROC curve; CTD: Comparative Toxicogenomics Database; GeneRIF: Gene Reference Into Function; GWAS: genome-wide association study; ID: identifier; MAP: mean average precision; MeSH: Medical Subject Heading; MeSHOP: Medical Subject Heading Over-representation Profile; NCBI: National Center for Biotechnology Information; NLM: US National Library of Medicine; OMIM: Online Mendelian Inheritance in Man; PCR: polymerase chain reaction; ROC: receiver operating characteristic.

## Competing interests

The authors declare that they have no competing interests.

## Authors' contributions

All authors contributed to the design of the method and the analysis and interpretation of the data. WAC implemented and carried out the study. All authors read and approved the final manuscript.
